# Prevalence and correlates of vitamin D deficiency in a mixed-age hospital-based cohort

**DOI:** 10.3389/fpubh.2026.1757957

**Published:** 2026-02-19

**Authors:** Zan Zhou, Si Chen, Fen Yang

**Affiliations:** Department of Laboratory Medicine, Liuyang Maternal and Child Health Hospital, Changsha, Hunan, China

**Keywords:** 25-hydroxyvitamin D, China, cross-sectional study, deficiency, prevalence, vitamin D

## Abstract

**Background:**

Vitamin D deficiency is a pervasive global health issue with significant implications for skeletal and extra-skeletal health. While its prevalence is well-documented in temperate climates, data from large, mixed-age hospital-based cohorts in subtropical regions of China remain limited. This study aimed to determine the precise prevalence and key correlates of vitamin D status in a large and diverse patient population to identify the most vulnerable subgroups.

**Methods:**

A retrospective, cross-sectional analysis was conducted on 22,484 valid serum 25-hydroxyvitamin D [25(OH)D] test results from patients at Liuyang Maternal and Child Health Care Hospital (March 2024–September 2025). Vitamin D status was categorized as deficient (≤ 20 ng/mL), low (21–29 ng/mL), or optimal (≥ 30 ng/mL). Group differences were assessed using chi-square tests, and correlations were evaluated with Spearman’s rank coefficient.

**Results:**

The overall prevalence of vitamin D deficiency was 10.3% (2,314/22,484), with 27.7% (6,221/22,484) low and 62.0% (13,949/22,484) optimal. The mean 25(OH)D concentration was 33.8 ± 11.9 ng/mL. A strong inverse correlation was observed between 25(OH)D and age (*ρ* = −0.351, *p* < 0.001). Deficiency rates varied markedly by age, being lowest in infants/neonates (3.1%) and young children (4.2%), but highest in adolescents (18.2%) and adults aged 18–39 (17.2–17.3%). Females had a significantly higher deficiency rate than males (13.1% vs. 5.8%, *p* < 0.001). A pronounced seasonal variation was evident, with deficiency peaking in spring (19.5%) and reaching its nadir in fall (4.5%). A weak inverse correlation was found between 25(OH)D and vitamin A (*ρ* = −0.036, *p* = 0.011).

**Conclusion:**

This large-scale hospital-based analysis reveals a significant burden of vitamin D deficiency, with a highly heterogeneous distribution across demographic and seasonal strata. The disproportionately high risk identified among adolescents, younger adults (particularly women), and during spring months underscores the critical need for targeted screening and intervention strategies for these specific vulnerable subgroups within similar clinical settings in subtropical China.

## 1 Introduction

Vitamin D, a fat-soluble secosteroid, is fundamentally involved in calcium and phosphate homeostasis, which is essential for bone health and mineralization (1, 2). Vitamin D deficiency affects an estimated 50% of the global population, with an estimated 1 billion people worldwide belonging to different ethnicities and age groups (3, 4). The risk factors for this worldwide deficiency included low dietary intake and decreased outdoor activities as well as environmental factors such as air pollution, which decreases the exposure to sunlight, therefore reducing the UVB-induced vitamin D synthesis (5). Vitamin D deficiency may cause secondary hyperparathyroidism, rickets, osteomalacia, osteoporosis, and even fragility fractures (6). In the past decade, vitamin D has also been investigated for a wide variety of extra-skeletal effects. While its role in immune regulation is well-supported, associations with conditions such as diabetes mellitus, cardiovascular disease, and cancer have shown inconsistent results in recent experimental studies, highlighting the need for further research (7–9). Many population-based studies on vitamin D deficiency have been conducted, however most have been performed in temperate countries with few being conducted in subtropical regions.

Vitamin D metabolism initiates with the cutaneous synthesis of pre-vitamin D from 7-dehydrocholesterol (7-DHC) following exposure to UVB radiation (280–320 nm), a process occurring predominantly in the basal layer of the epidermis (10). Inactive vitamin D3 (endogenous) and vitamin D2 or D3 (dietary) are subsequently absorbed and transported via circulation, bound primarily to Vitamin D Binding Protein (VDBP), a member of the albumin superfamily synthesized in the liver (11, 12). To exert its hormonal function, vitamin D requires two sequential hydroxylation steps (13). The first hydroxylation, catalyzed by 25-hydroxylase in the liver, yields 25-hydroxyvitamin D3 [25(OH)D3], which serves as the principal indicator of vitamin D status in humans (14). The second hydroxylation occurs in the renal proximal tubules, mediated by 1α-hydroxylase (CYP27B1), resulting in the biologically active hormone, calcitriol [1,25(OH)2D3] (15). Calcitriol binds to the Vitamin D Receptor (VDR) to modulate gene expression, thereby regulating calcium homeostasis by enhancing intestinal and renal calcium absorption and increasing osteoclast activity (16).

Vitamin D deficiency has been reported to be more common than previously thought, and it has become a public health issue in modern societies (17). The prevalence of vitamin D deficiency varies significantly in different countries and populations (18), investigating the prevalence and associated sociodemographic factors of vitamin D deficiency in subtropical areas is needed. In the present retrospective, cross-sectional analysis, we evaluated 22,484 unique serum 25-hydroxyvitamin D [25(OH)D] test results obtained between March 2024 and September 2025 at Liuyang Maternal and Child Health Care Hospital, a tertiary institution in China, to examine the prevalence and heterogeneity of vitamin D status across age, sex, department, and season in this large mixed-age hospital-based cohort.

## 2 Materials and methods

### 2.1 Study design and population

This retrospective, cross-sectional study evaluated the prevalence and variation of serum 25-hydroxyvitamin D [25(OH)D] concentrations in a heterogeneous patient population from Changsha, China. Data were obtained from the Central Clinical Laboratory of Liuyang Maternal and Child Health Care Hospital, a tertiary academic institution serving both urban and suburban communities. All biochemical test results performed between March 2024 and September 2025 were extracted from the hospital’s electronic Laboratory Information System (LIS). This 18-month interval was selected to capture samples across all four meteorological seasons, thereby enabling the assessment of seasonal variation in vitamin D status. The study’s analytical framework was adapted from methods used in similar regional assessments, such as those conducted in Western Romania (19) and Northern China (20).

### 2.2 Inclusion and exclusion criteria

**Figure 1:**
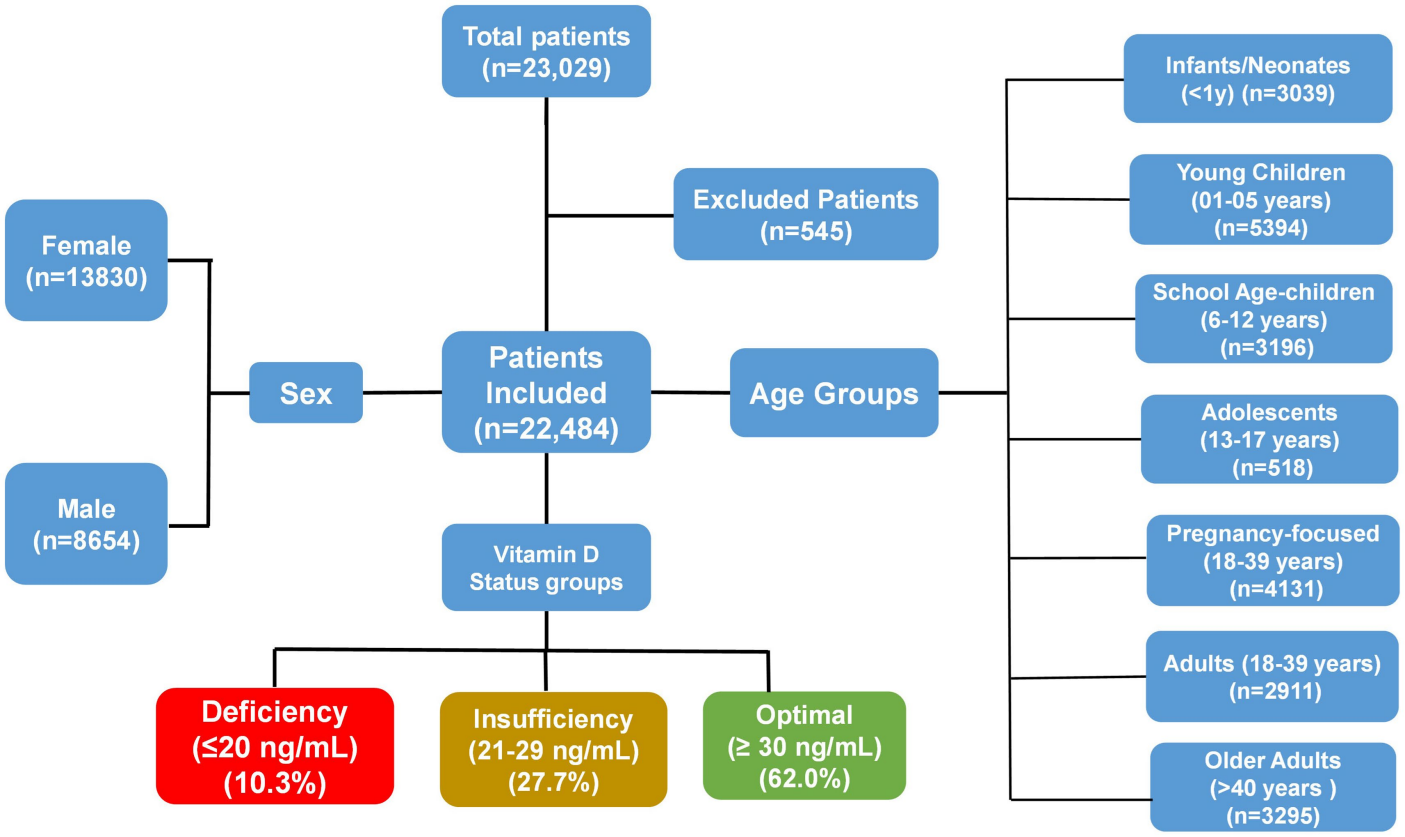
Flowchart detailing patient distribution and vitamin D status.

A total of 23,029 records were initially retrieved from the LIS. After rigorous data cleaning, unique cases were retained, of which 22,484 valid 25(OH)D determinations were available for statistical analysis. Inclusion criteria were: (1) complete demographic and biochemical data, including test date, age, gender, ordering department, and total 25(OH)D concentration; and (2) laboratory records within the defined sampling window (March 2024–September 2025). Exclusion criteria included missing demographic information (~545 entries), duplicate test identifiers (none detected), or physiologically implausible values (Figure 1).

Participants were stratified into seven age-related groups: infants/neonates (< 1 year), young children (1–5 years), school-age children (6–12 years), adolescents (13–17 years), adults aged 18–39 years in pregnancy-focused departments, adults aged 18–39 years in non-pregnancy departments, and older adults (> 40 years). These categories were adapted from prior pediatric and adult vitamin D studies in Asia and Europe (19). Hospital departments were aggregated into five categories; Neonatology, Children’s Health, Pre-Pregnancy, Adult Comprehensive, and Other to facilitate subgroup analyses by clinical setting.

### 2.3 Sample processing and laboratory measurements

All blood specimens were processed by the hospital’s accredited clinical laboratory under standardized operating procedures. Serum 25(OH)D and Vitamin A concentrations were measured using a two-dimensional liquid chromatography (2D-LC) (Fully Automatic 2D Liquid Chromatography Analysis System, Hunan Aidaimai Biotechnology Co., Ltd., QG3000). The analyzer simultaneously quantified 25(OH)D₂ and 25(OH)D₃, and their sum was recorded as the total 25(OH)D concentration. Assay performance was verified through internal quality control procedures, yielding intra-assay and inter-assay coefficients of variation < 5% and < 10%, respectively. All analyses adhered to the manufacturer’s specifications and the laboratory’s internal quality standards. Vitamin D status was classified in accordance with Endocrine Society and Chinese Nutrition Society criteria: Deficient: ≤ 20 ng/mL; Low: 21–29 ng/mL; Optimal: ≥ 30 ng/mL. These thresholds are consistent with internationally accepted reference intervals and comparable with previous reports (20, 21).

### 2.4 Variable definitions and seasonal classification

The following variables were extracted for each record: test date, gender, patient age, ordering department, 25(OH)D₂, 25(OH)D₃, total 25(OH)D, and vitamin A concentration (where available). Seasonal classification was based on the test date: Spring (March–May), Summer (June–August), Fall (September–November), and Winter (December–February), following established frameworks for vitamin D epidemiology in subtropical climates. Yearly comparisons (2023 vs. 2025) were evaluated to identify temporal consistency, although the two-year interval was not sufficient for formal longitudinal trend analysis.

### 2.5 Statistical analysis

All statistical analyses were performed using R version 4.3.1 (R Foundation for Statistical Computing, Vienna, Austria). Continuous variables were expressed as mean ± standard deviation (SD), and categorical variables as counts and percentages. Normality was assessed using the Shapiro–Wilk test. Between-group comparisons were conducted using Student’s t-test or one-way ANOVA, as appropriate. Associations between categorical variables (vitamin D status by age, gender, department, and season) were analyzed using chi-square (*χ*^2^) tests. Significant group differences were confirmed as follows: *χ*^2^ (6 df) = 2383.2, *p* < 0.001 for age group; *χ*^2^ (8 df) = 1398.5, p < 0.001 for department group; *χ*^2^ (2 df) = 536.0, *p* < 0.001 for gender; *χ*^2^ (6 df) = 1253.9, *p* < 0.001 for season. Spearman’s rank correlation analysis evaluated relationships between continuous variables: 25(OH)D vs. age: *p* = −0.351, *p* < 0.001; 25(OH)D vs. vitamin A: *p* = −0.036, *p* = 0.011. A two-tailed *p* < 0.05 was considered statistically significant.

### 2.6 Ethical considerations

This study complied with the ethical principles of the Declaration of Helsinki and has been approved by the Ethics Committee of Liuyang Maternal and Child Health Care Hospital (HL20230115).

## 3 Results

### 3.1 Participant characteristics

**Figure 2:**
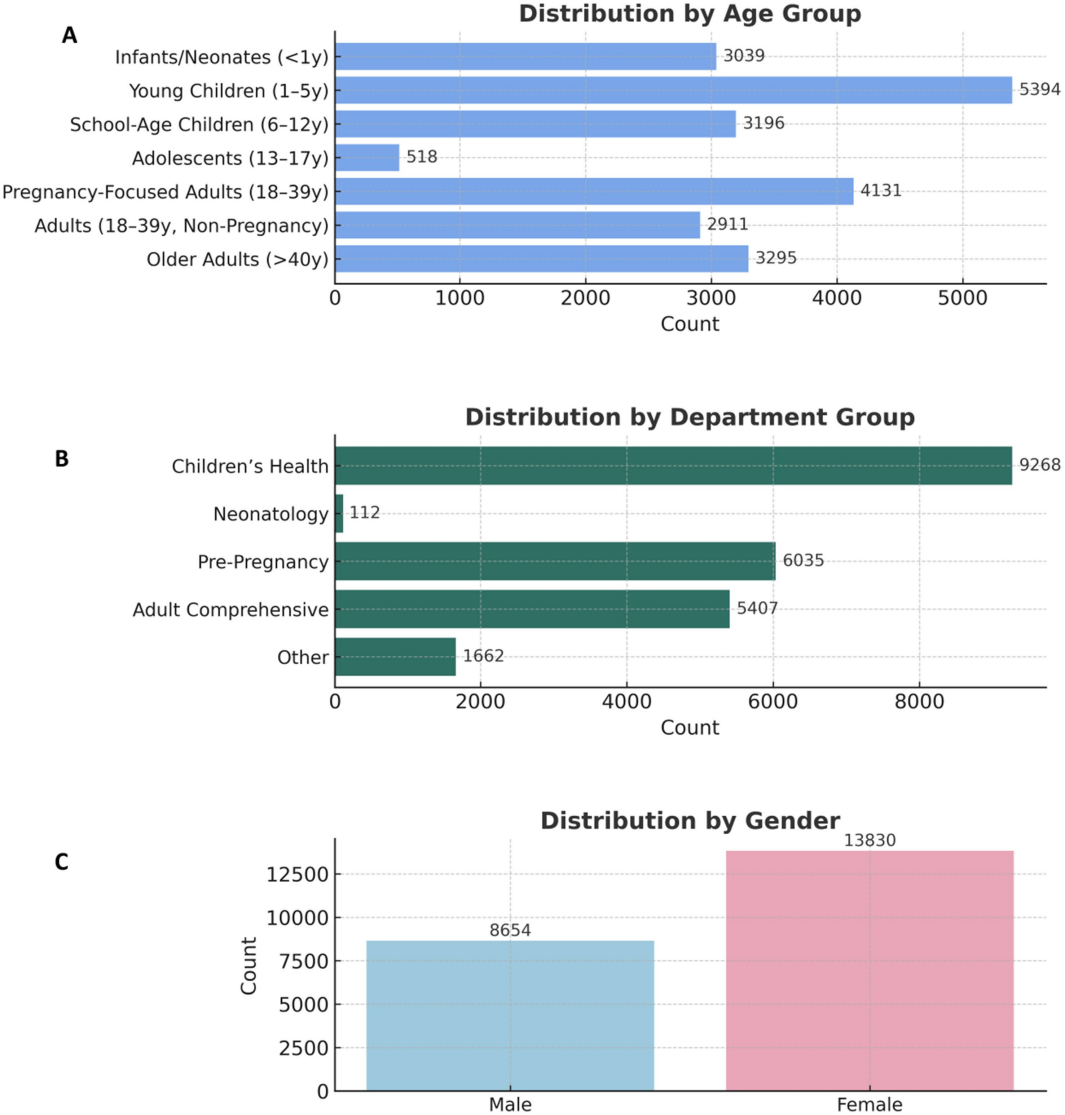
Demographic and departmental characteristics of study participants **(A)** Distribution of participants by age group (*n* = 22,484). Age categories were defined as: infants/neonates (<1 year; 13.5%, *n* = 3,039), young children (1–5 years; 24.0%, *n* = 5,394), school-age children (6–12 years; 14.2%, *n* = 3,196), adolescents (13–17 years; 2.3%, *n* = 518), pregnancy-focused adults (18–39 years; 18.4%, *n* = 4,131), adults aged 18–39 years from non-pregnancy departments (12.9%, *n* = 2,911), and older adults (>40 years; 14.7%, *n* = 3,295). **(B)** Distribution by clinical department of 25-hydroxyvitamin D [25(OH)D] test orders (*n* = 22,484). Most measurements originated from Children’s Health (41.4%, n=9,268) and Adult Comprehensive (24.1%, *n* = 5,407) departments, followed by Pre-Pregnancy (26.9%, *n* = 6,035), Other (7.4%, *n* = 1,662), and Neonatology (0.5%, *n* = 112) units. **(C)** Female participants predominated (61.5%, *n* = 13,830) compared with males (38.5%, n=8,654), consistent with the hospital’s maternal and pediatric care focus.

A total of 22,484 participants from Liuyang Maternal and Child Health Care Hospital, located in Changsha, China, were included in the final analysis following rigorous quality control and exclusion of incomplete entries. Participants spanned a wide age range, from newborns to 95 years, reflecting the hospital’s mixed pediatric, obstetric, and adult service profile. However, the low mean age (18.4 ± 15.7 years) and median age (12 years) underscore that the hospital-based cohort is predominantly composed of children and young adults, which is consistent with the hospital’s specialized focus on maternal and child health. Females accounted for the majority of participants (*n* = 13,187; 58.6%), consistent with the hospital’s focus on maternal and child health services. The age-group distribution was as follows: infants/neonates (< 1 year): 13.5% (*n* = 3039); young children (1-5 years): 24.0% (*n* = 5394); school-age children (6-12 years): 14.2% (*n* = 3196); adolescents (13-17 years): 2.3% (*n* = 518); pregnancy-focused adults (18-39 years): 18.4% (*n* = 4 131); non-pregnant adults (18-39 years): 12.9% (*n* = 2911); and older adults (> 40 years): 14.7% (*n* = 3295) (Figure 2).

The majority of 25-hydroxyvitamin D [25(OH)D] measurements were ordered from Children’s Health (*n* = 9,268; 41.2%) and Adult Comprehensive (*n* = 5,407; 24.0%) departments, followed by Pre-Pregnancy (*n* = 6,035; 26.9%), Other (*n* = 1,662; 7.4%), and Neonatology (*n* = 112; 0.5%) units. The total sample size for departmental analysis was *N* = 22,484 (Figure 2B). These distributions mirror the hospital’s integrated maternal and pediatric care model. Sample collection occurred continuously across all four seasons, with a modest predominance of summer and fall samples, consistent with the study’s 18-month sampling period (March 2024 - September 2025).

### 3.2 Serum vitamin D status in the study population

**Figure 3:**
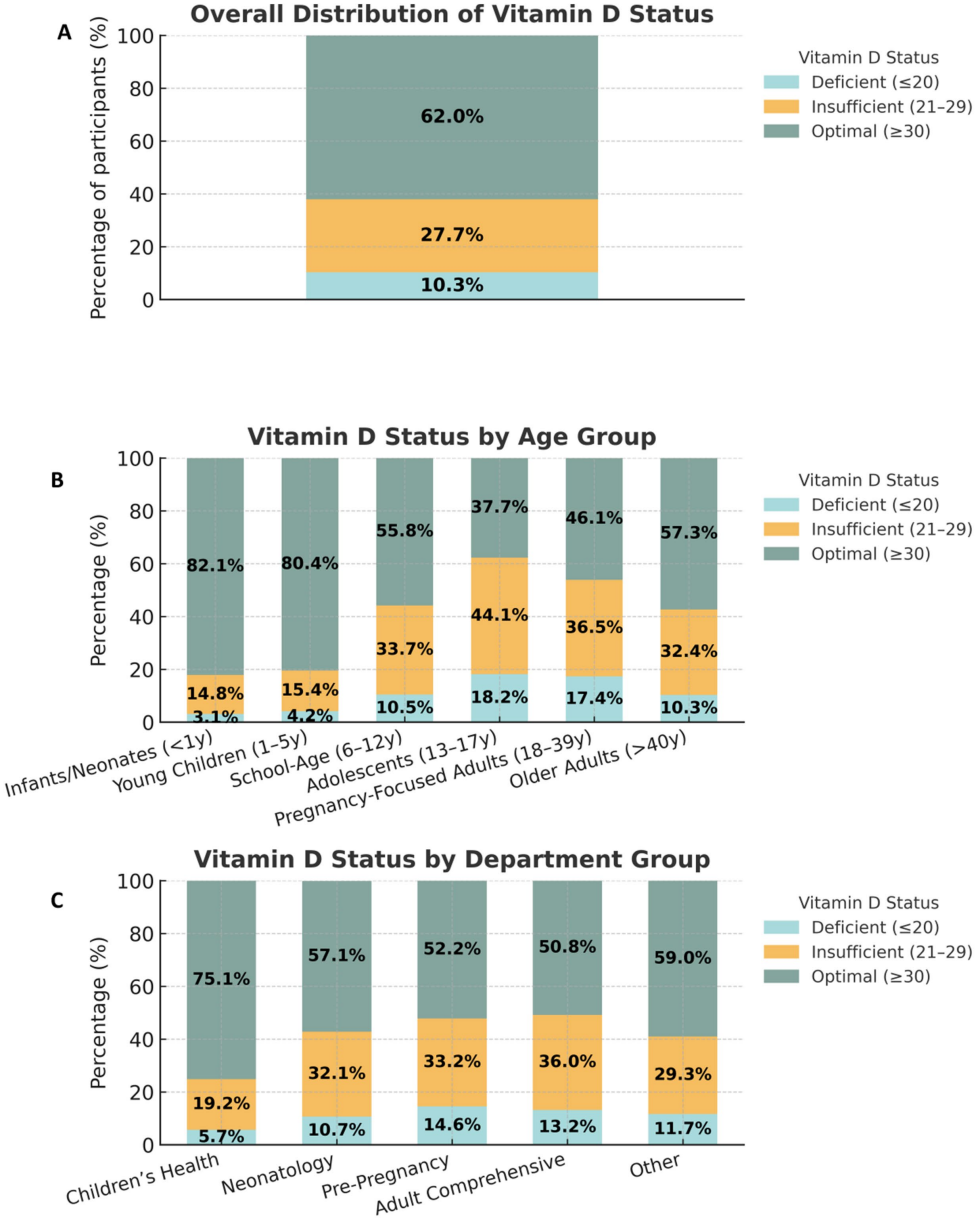
Distribution of serum 25-hydroxyvitamin D (25(OH)D) **(A)** Overall distribution of vitamin D status in the total cohort (*n* = 22,484). A majority of participants (62.0%) had optimal levels (≥30 ng/mL), while 27.7% were insufficient (21–29 ng/mL), and 10.3% were deficient (≤20 ng/mL). **(B)** Age-stratified prevalence of vitamin D status categories. Vitamin D sufficiency was highest in infants (<1y) and young children (1–5 y), 82.1% and 80.4% optimal levels, respectively. A consistent decline in optimal status was observed with increasing age, with adolescents (13–17 y) having the lowest pediatric sufficiency (37.7%). Adults, particularly pregnancy-focused women, showed optimal values (46.1%). **(C)** Department-stratified prevalence of vitamin D status. The most favorable profile was observed in the Children’s Health department (75.1% optimal, 5.7% deficient). In contrast, the Adult Comprehensive and Pre-Pregnancy departments showed the highest burden of deficiency (13.2% and 14.6%, respectively) and the lowest prevalence of optimal status (50.8% and 52.2%, respectively).

Serum 25-hydroxyvitamin D [25(OH)D] concentrations displayed wide interindividual variation, reflecting diverse vitamin D status among individuals tested at Liuyang Maternal and Child Health Care Hospital between March 2024 and September 2025. Overall, 62.0% of participants exhibited optimal 25(OH)D levels (≥ 30 ng/mL), whereas 27.7% were low (21–29 ng/mL) and 10.3% were deficient (≤ 20 ng/mL) (Figure 3A). These findings indicate that most individuals achieved adequate vitamin D status; however, a substantial minority remained below recommended thresholds.

Age-stratified analysis revealed pronounced developmental differences (Figure 3B). Infants and neonates (<1 year) exhibited the highest prevalence of optimal 25(OH)D levels (82.1%; 2,495/3,039), followed by young children aged 1–5 years (80.4%; 4,337/5,394). Prevalence declined with increasing age, falling to 55.8% (1,783/3,196) in school-age children (6–12 years) and reaching the lowest observed level of 37.7% (195/518) in adolescents (13–17 years). Among adults, pregnancy-focused women (18–39 years) showed 46.1% optimal status (1,904/4,131), while non-pregnant adults aged 18–39 years showed a comparable prevalence (46.3%; 1,347/2,911). Older adults (>40 years) demonstrated a higher proportion of optimal levels (57.3%; 1,888/3,295) (Figure 3B). Sex-stratified comparisons indicated higher optimal prevalence in males than females in infancy (82.8% vs 81.5%), young children (82.5% vs 78.5%), and school-age children (62.0% vs 50.0%); in adolescents, females showed slightly higher optimal prevalence than males (40.8% vs 32.9%). Across pediatric strata, deficiency was consistently more common in females than males (e.g., school-age children: 15.0% vs 10.9%), and this pattern persisted in older adults (9.8% vs 7.5%). Pregnancy-focused women also exhibited a notable burden of deficiency (15.3%). Overall, these results highlight age- and sex-related disparities in vitamin D status (Table 1).

Departmental comparisons further substantiated these trends (Figure 3C). The Children’s Health showed the most favorable distribution, where 75.1% of participants achieved an optimal vitamin D level (≥30 ng/mL). In contrast, other departments had a significantly lower prevalence of optimal status, ranging from 50.8% in Adult Comprehensive to 57.1% in Neonatology. Concurrently, the prevalence of deficiency (≤20 ng/mL) was lowest in the Children’s Health group (5.7%). The highest deficiency rates were observed in the Pre-Pregnancy (14.6%) and Adult Comprehensive (13.2%) groups. The Neonatology department had an intermediate deficiency rate of 10.7%. The prevalence of insufficiency (21–29 ng/mL) was also most favorable in the Children’s Health department (19.2%), while it exceeded 30% in the Pre-Pregnancy (33.2%) and Adult Comprehensive (36.0%) groups. These findings indicate that vitamin D sufficiency is significantly higher in the pediatric population served by the Children’s Health department, while adult-focused and pre-pregnancy care groups exhibit a greater burden of vitamin D deficiency and insufficiency.

**Table 1:** Prevalence of vitamin D status by gender within age groups (*n*, %).

Age group	Gender	Deficient (*n*, %)	Low (*n*, %)	Optimal (*n*, %)
Infants/Neonates (<1y)	Female	131 (5.8)	26 (1.2)	2,099 (93.2)
	Male	99 (4.4)	18 (0.8)	2,139 (96.2)
Young Children (1-5y)	Female	142 (4.2)	783 (23.1)	2,579 (76.1)
	Male	88 (2.6)	651 (19.1)	2,546 (80.3)
School-Age Children (6-12y)	Female	342 (19.8)	635 (36.7)	756 (43.7)
	Male	266 (15.4)	521 (30.1)	936 (53.9)
Adolescents (13-17y)	Female	81 (13.2)	231 (37.6)	302 (49.2)
	Male	59 (9.6)	201 (32.8)	173 (57.6)
Pregnancy-Focused (18-39y)	Female	790 (17.3)	1,580 (34.6)	2,197 (48.1)
	Male	N/A	N/A	N/A
Older Adults (>40y)	Female	208 (10.8)	623 (32.3)	1,045 (54.2)
	Male	150 (7.8)	482 (25.0)	1,164 (60.4)

### 3.3 Seasonal variation in vitamin D status

**Figure 4:**
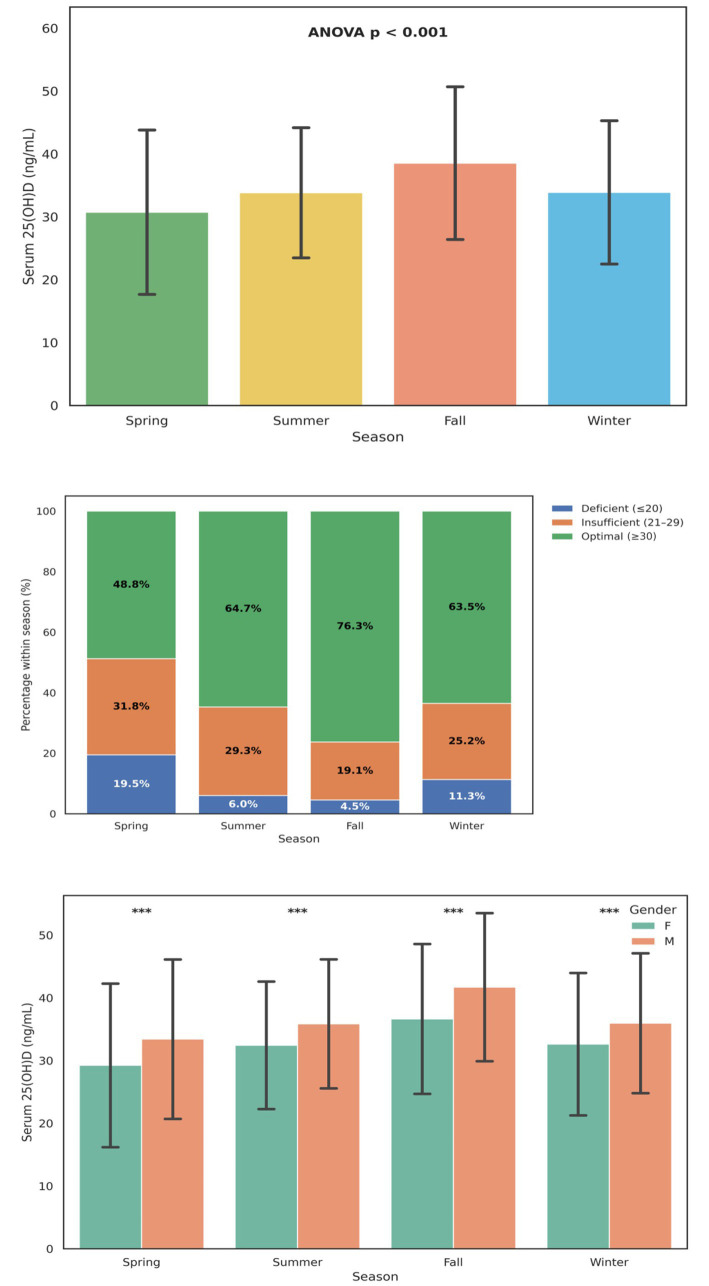
Seasonal variation in serum 25-hydroxyvitamin D [25(OH)D] levels and deficiency prevalence. **(A)** Mean ± SD serum 25(OH)D concentrations across the four seasons (Spring, Summer, Fall, Winter). A significant difference in mean 25(OH)D levels was observed among seasons (ANOVA *p* < 0.001). **(B)** Proportional distribution of vitamin D status; deficient (≤ 20 ng/mL), low (21–29 ng/mL), and optimal (≥ 30 ng/mL). The prevalence of deficiency was highest in Spring (19.5%) and lowest in Fall (4.5%), with optimal vitamin D levels most frequent during Fall (76.3%). **(C)** Seasonal variation in mean 25(OH)D concentrations stratified by gender (Mean ± SD). Across all seasons, male participants exhibited significantly higher 25(OH)D levels than females (*p* < 0.001). Error bars represent standard deviations. Percentages are displayed within each bar segment.

Serum 25(OH)D levels exhibited significant seasonal fluctuations among the study population (ANOVA *p* < 0.001), indicating that environmental sunlight exposure and related behavioral factors strongly influenced circulating vitamin D concentrations (Figure 4A). The mean ± SD 25(OH)D concentrations increased progressively from Spring (30.5 ± 13.8 ng/mL) to Fall (38.6 ± 12.8 ng/mL) before declining slightly in Winter (33.9 ± 12.9 ng/mL). This pattern reflects the cumulative effect of greater ultraviolet B (UVB) exposure during summer and autumn months, consistent with trends reported in East Asian and European populations.

The distribution of vitamin D status categories also varied markedly by season (Figure 4B). The proportion of vitamin D deficiency (≤ 20 ng/mL) peaked in Spring (19.5%), declined sharply during Summer (6.0%) and Fall (4.5%), and rose moderately in Winter (11.3%). Conversely, the prevalence of optimal vitamin D levels (≥ 30 ng/mL) was highest during Fall (76.3%) and lowest in Spring (48.8%). These findings confirm a pronounced seasonal rhythm in vitamin D sufficiency, with maximum adequacy following summer sunlight exposure and transient declines during colder months. When stratified by gender (Figure 4C), men consistently demonstrated higher 25(OH)D concentrations than women across all four seasons (*p* < 0.001).

Table 2 summarizes seasonal variation in vitamin D status across all participants. Optimal 25(OH)D levels peaked during the fall (76.3%) and summer (64.7%), while spring had the highest proportion of deficiency (19.5%). Winter values were intermediate, consistent with reduced sunlight exposure but possible supplementation carryover. These results align with the patterns illustrated in Figure 3 and confirm significant seasonal effects (*χ*^2^ = 1,253.9, *p* < 0.001). Supplementary Table S1 further stratifies vitamin D deficiency rates by both season and age group. The greatest seasonal fluctuation occurred in school-age children and pregnancy-focused adults, in whom deficiency rose sharply during spring (25.3 and 24.8%, respectively) and declined markedly in fall (6.5 and 8.7%). In contrast, infants and young children maintained stable sufficiency year-round. These data underscore differential seasonal vulnerability among older pediatric and adult subgroups. This gender disparity likely reflects combined effects of occupational sunlight exposure, outdoor activity levels, and physiological differences in vitamin D metabolism. Together, these seasonal and gender-related trends underscore the influence of environmental and demographic factors on vitamin D homeostasis in the population.

**Table 2:** Prevalence of vitamin D status by season (*n*, %).

Season	VitD_Status	*n*	Percentage
Spring	Deficient	1,212	19.5
Spring	Low	1979	31.8
Spring	Optimal	3,038	48.8
Summer	Deficient	550	6
Summer	Low	2,678	29.3
Summer	Optimal	5,923	64.7
Fall	Deficient	168	4.5
Fall	Low	707	19.1
Fall	Optimal	2,819	76.3
Winter	Deficient	384	11.3
Winter	Low	857	25.2
Winter	Optimal	2,163	63.5

### 3.4 Association of Vitamin D Status with age group and clinical department

Analysis of serum 25(OH)D concentrations across different age categories revealed a pronounced age-dependent gradient in vitamin D status (*p* < 0.001; Figure 3B). The highest mean 25(OH)D levels were observed among infants and neonates (< 1 year; 82.1% optimal) and young children aged 1-5 years (80.4% optimal), reflecting widespread supplementation practices and regular monitoring in early life. In contrast, vitamin D sufficiency progressively declined with increasing age: school-age children (6–12 years) demonstrated 55.8% optimal, while adolescents (13–17 years) exhibited the lowest sufficiency (37.7%). Among adults, older adults (>40 years) showed a higher proportion of optimal levels (57.3%) than pregnancy-focused adults (18–39 years; 46.1%).

When analyzed by clinical department (Figure 3C), the distribution of vitamin D status differed significantly across patient categories (χ^2^ *p* < 0.001). The Children’s Health department demonstrated the highest prevalence of optimal vitamin D levels (75.1%), followed by Neonatology (57.1%), reflecting strong adherence to preventive pediatric supplementation and nutritional guidelines. In contrast, the Pre-Pregnancy and Adult Comprehensive departments exhibited higher rates of deficiency (14.6 and 13.2%, respectively), consistent with limited vitamin D awareness and reduced routine screening among adults. Patients categorized under other departments (13.0%) displayed heterogeneous results due to diverse clinical indications for testing.

Collectively, these findings underscore the strong influence of developmental stage and clinical specialization on vitamin D status within the population. Pediatric and maternal groups benefit from structured supplementation programs and regular testing, while adolescents and non-pregnant adults remain vulnerable to sub-optimal vitamin D levels due to lower healthcare engagement and lifestyle factors such as reduced outdoor exposure. These demographic disparities justify targeted public health interventions aimed at improving vitamin D awareness and screening among adult and adolescent populations.

### 3.5 Relationship between vitamin a and vitamin D status

**Figure 5:**
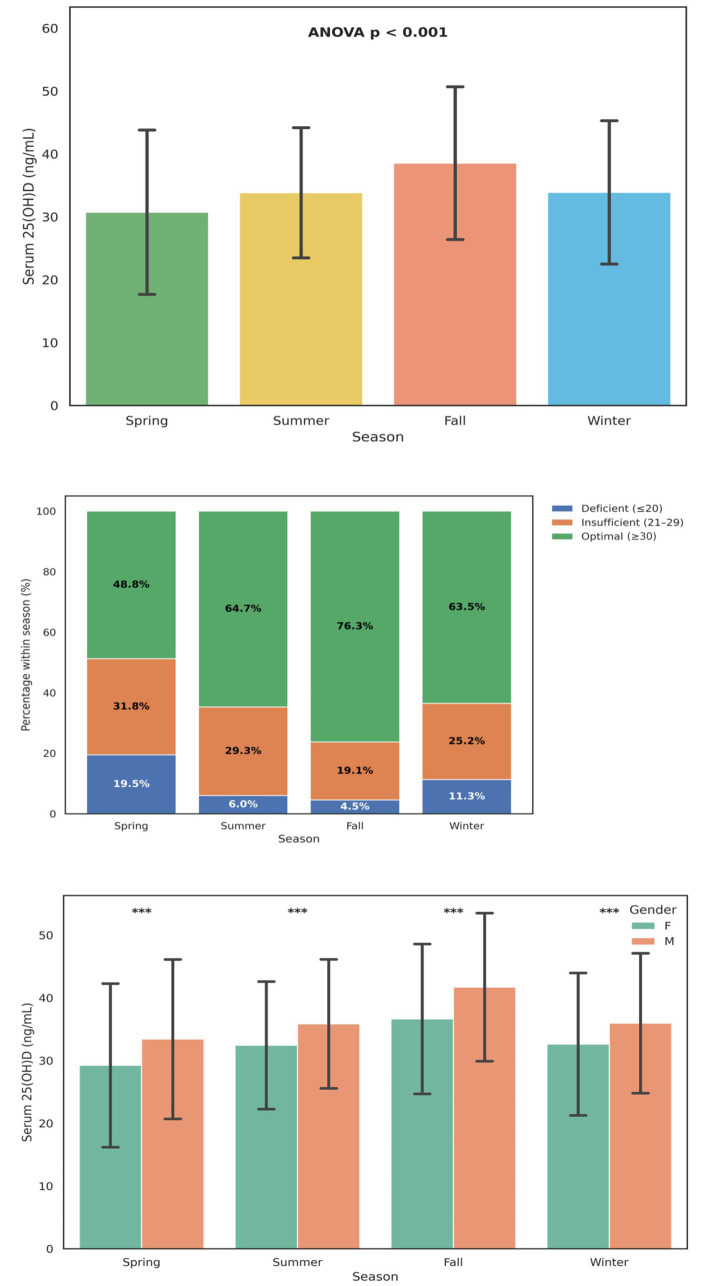
Association between serum vitamin A levels and vitamin D status. **(A)** The correlation between serum vitamin A and 25-hydroxyvitamin D [25(OH)D] concentrations among study participants. A weak inverse relationship was observed (Pearson r = –0.05, *p* = 0.00). **(B)** Vitamin A levels across vitamin D status categories. Median vitamin A levels were comparable among the deficient (≤ 20 ng/mL), low (21-29 ng/mL), and optimal (≥ 30 ng/mL) vitamin D groups, with modest variability reflected by overlapping interquartile ranges. **(C)** vitamin D status across tertiles of serum vitamin A. Participants with low (T1), mid (T2), and high (T3) vitamin A levels exhibited similar proportions of vitamin D sufficiency. Percentages represent within tertile proportions.

To explore potential biochemical interplay between fat-soluble vitamins, serum vitamin A concentrations were analyzed in relation to circulating 25(OH)D levels. As shown in Figure 5A, correlation analysis revealed a weak but statistically significant negative association between vitamin A and 25(OH)D (Pearson r = −0.05, *p* = 0.00), suggesting minimal inverse coupling between these nutrients. Comparison across vitamin D status groups (Figure 5B) showed no meaningful trend in median vitamin A levels among deficient, low, or optimal categories (*p* > 0.05 by ANOVA). Stratification by vitamin A tertiles (Figure 5C) further confirmed the absence of major shifts in vitamin D sufficiency prevalence across increasing vitamin A concentrations. Collectively, these findings indicate that within this population, vitamin A status exerts little measurable influence on serum 25(OH)D concentrations.

### 3.6 Associations between serum 25-hydroxyvitamin D and biochemical parameters (NHANES 2009–2012)

**Figure 6:**
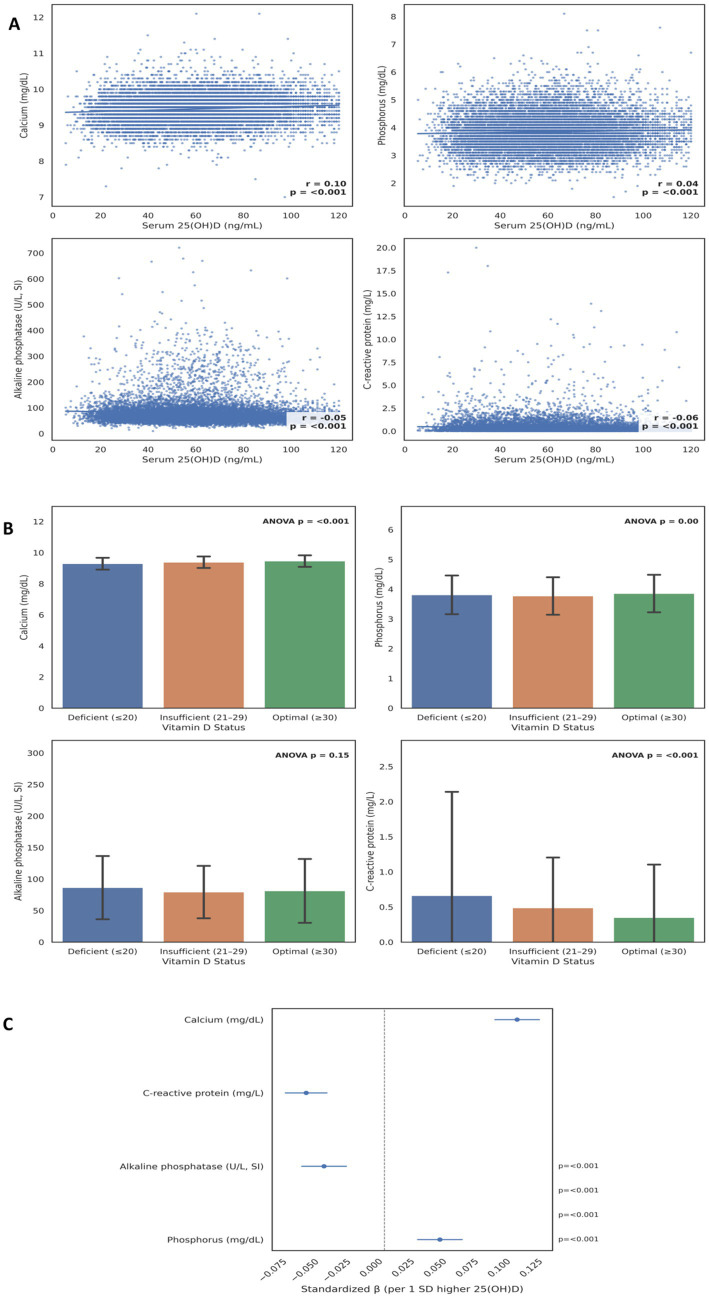
Biochemical and inflammatory correlates of serum 25-hydroxyvitamin D in the NHANES 2009-2012 dataset. **(A)** Pearson correlations between serum 25-hydroxyvitamin D [25(OH)D] and key biochemical markers, including calcium, phosphorus, alkaline phosphatase (ALP), and C-reactive protein (CRP). Correlation coefficients (r) and p values are indicated within each panel. **(B)** Mean ± SD levels of biochemical parameters across vitamin D status categories; deficient (≤20 ng/mL), low (21-29 ng/mL), and optimal (≥30 ng/mL). **(C)** Standardized β coefficients (Cl 95%) from multi-variable linear models adjusted for survey cycle. Positive β values indicate direct associations with 25(OH)D, while negative β values denote inverse relationships.

To extend the biochemical insights observed in our Changsha hospital-based cohort (Section 3.4), we validated these findings using data from the U. S. National Health and Nutrition Examination Survey (NHANES 2009–2012). This nationally representative dataset includes standardized assays for 25-hydroxyvitamin D [25(OH)D], calcium, phosphorus, alkaline phosphatase (ALP), and C-reactive protein (CRP)—parameters that reflect bone-mineral metabolism and systemic inflammation. By analyzing these variables across two consecutive survey cycles, we sought to determine whether the associations identified in the Chinese population were consistent at the population level. As shown in Figure 6A, serum 25(OH)D levels were positively correlated with calcium (*r* = 0.10, *p* < 0.001) and phosphorus (*r* = 0.04, *p* < 0.001), and inversely correlated with ALP (*r* = −0.05, *p* < 0.001) and CRP (*r* = −0.06, *p* < 0.001). Although modest in magnitude, these correlations were highly significant, suggesting a consistent biochemical link between vitamin D sufficiency, mineral balance, and reduced inflammatory activity (Supplementary Table S2).

Group-wise comparisons further demonstrated that participants with optimal vitamin D status (≥30 ng/mL) exhibited higher mean calcium and phosphorus levels (ANOVA *p* < 0.001 for both) and lower CRP concentrations (ANOVA *p* < 0.001) compared with those who were deficient or low (Figure 6B). ALP activity did not differ significantly among categories (ANOVA *p* = 0.15). This dose–response pattern indicates that physiological benefits of vitamin D become more apparent as serum concentrations approach sufficiency thresholds. In multivariable models adjusted for survey cycle, each one-standard-deviation increase in serum 25(OH)D was associated with higher calcium (*β* = +0.10, 95% CI 0.09–0.12, *p* < 0.001) and phosphorus (*β* = +0.04, 0.03–0.06, *p* < 0.001), and lower ALP (*β* = −0.05, −0.07 to −0.03, *p* < 0.001) and CRP (*β* = −0.06, −0.08 to −0.05, *p* < 0.001) (Figure 6C). These associations persisted after adjusting for inter-cycle variation, supporting their reproducibility across different sampling periods.

Taken together, the NHANES analyses corroborate the biochemical patterns identified in our Changsha hospital-based cohort. Across geographically and ethnically distinct populations, optimal vitamin D status consistently aligns with favorable calcium-phosphate regulation and reduced inflammatory burden. This convergence underscores the biological universality of vitamin D’s role in maintaining mineral and immune homeostasis.

## 4 Discussion

In this large-scale, retrospective cross-sectional study conducted at a major maternal and child health institution in Eastern Hunan, China, we delineated the intricate landscape of vitamin D status across a mixed-age hospital-based cohort of 22,484 individuals. Our analysis reveals three central findings: first, a significant burden of vitamin D deficiency and insufficiency that is not uniformly distributed but is concentrated in specific demographic subgroups; second, a pronounced seasonal rhythm in serum 25(OH)D concentrations; and third, a consistent association between vitamin D sufficiency and favorable biochemical profiles, a relationship validated in an independent, geographically distinct population from the NHANES dataset. The integration of our local data with this external hospital-based cohort strengthens the biological plausibility and generalizability of our conclusions.

The overall prevalence of vitamin D deficiency (10.3%) and insufficiency (27.7%) in our hospital-based cohort is substantial, yet it reveals a more nuanced picture than previously reported in some Chinese populations (22, 23). The relatively high mean 25(OH)D concentration (33.8 ± 11.9 ng/mL) and the majority (62.0%) of participants achieving optimal status are encouraging, likely reflecting successful public health supplementation programs targeting infants and young children, as evidenced by the exceptionally high sufficiency rates in these groups (>80%). However, this overall adequacy masks critical vulnerabilities. The most striking finding was the strong inverse correlation between age and 25(OH)D levels (*ρ* = −0.351, *p* < 0.001), with deficiency rates escalating from a low of 3.1% in infants to a peak of 18.2% in adolescents. This pattern aligns with studies from Romania and other regions, which also note a decline in vitamin D status with advancing age from childhood into adolescence and young adulthood (19, 24). The biological and behavioral rationale for this disparity is multifactorial. Infants and young children benefit from routine vitamin D supplementation protocols and close nutritional supervision. In contrast, adolescents experience a perfect storm of risk factors: a period of rapid skeletal growth that dramatically increases physiological demand, coupled with behavioral shifts toward sedentary, indoor lifestyles, increased academic pressure, and potentially inadequate dietary intake (24). For adults aged 18–39, particularly women in pregnancy-focused care, the high deficiency rate (17.2–17.3%) can be attributed to factors such as indoor occupations, cosmetic use of sunscreen, and the physiological drain of pregnancy on maternal vitamin D stores to support fetal skeletal development (25, 26). The significant gender disparity, with females having more than double the deficiency rate of males (13.1% vs. 5.8%), further underscores the heightened risk for women of reproductive age, potentially linked to hormonal factors and differences in sun-avoidance behaviors.

The observed seasonal variation in 25(OH)D levels is a classic phenomenon, yet its pattern in our subtropical hospital-based cohort provides critical public health insights. The nadir of deficiency in the fall (4.5%) represents the cumulative benefit of summer sun exposure, while the peak in spring (19.5%) reflects the depletion of vitamin D stores over the winter months. This pattern is consistent with the well-established dependence of cutaneous vitamin D synthesis on the intensity of solar UVB radiation, which is significantly diminished during winter (27). However, the pronounced spring peak, despite increasing daylight hours, suggests a lag effect and may be exacerbated by behavioral factors such as the continued wearing of longer sleeves in the cooler early spring. This finding provides a strong physiological rationale for a targeted, seasonally-timed intervention strategy. Rather than generalized year-round supplementation for all, our data suggest that initiating or intensifying vitamin D supplementation at the end of winter could be a more efficient public health approach to pre-empt the spring nadir and maintain year-round sufficiency in at-risk populations.

Our analysis of the relationship between vitamin A and D status revealed a weak but statistically significant inverse correlation (*ρ* = −0.036, *p* = 0.011). While this finding is intriguing, the very low correlation coefficient and the absence of a clear dose–response relationship across vitamin A tertiles suggest that its clinical relevance in this hospital-based cohort is minimal. The interplay between these fat-soluble vitamins is complex and reported in the literature as sometimes conflicting, potentially involving competitive absorption or shared metabolic pathways. In the context of our population, vitamin A status does not appear to be a major determinant of vitamin D concentration.

To extend the biochemical insights beyond associative patterns, we validated our findings using the NHANES 2009–2012 dataset. The consistent, albeit modest, positive correlations between 25(OH)D and serum calcium (r = 0.10) and phosphorus (r = 0.04), and the inverse correlations with ALP (r = −0.05) and CRP (r = −0.06), provide robust external confirmation of vitamin D’s fundamental role in mineral homeostasis and immune regulation (28, 29). The dose–response relationship observed in NHANES, where optimal vitamin D status was associated with more favorable biochemical profiles, underscores the physiological benefits of maintaining 25(OH)D levels above the sufficiency threshold. This convergence of findings across ethnically and geographically distinct populations highlights the biological universality of vitamin D’s functions and strengthens the causal inference from our primary data.

The strengths of this study include its very large sample size, the mixed-age design capturing a unique population from infancy to older adulthood, multi-seasonal assessment, and the unique methodological strength of validating biochemical associations in an independent international hospital-based cohort. However, several important limitations that should inform the interpretation of its findings. The retrospective, single-center, hospital-based design inherently limits the generalizability of our prevalence estimates to the broader community, as the hospital-based cohort consists of individuals for whom testing was clinically indicated. Methodologically, vitamin D status was assessed using a CLIA platform. Although our laboratory maintained excellent precision (CV < 10%), immunoassays can demonstrate systematic differences from reference LC–MS/MS methods, a factor to consider when comparing absolute 25(OH)D values across studies. A significant constraint is the lack of data on major determinants of vitamin D status. We could not adjust for BMI (a strong inverse correlate), measure PTH (needed to define a functional sufficiency threshold), or account for behavioral factors like time outdoors, clothing, or sunscreen use. These unmeasured confounders limit causal inference regarding the identified risk factors. Furthermore, the external validation using NHANES 2009–2012 data confirms persistent biochemical relationships but may not capture contemporary trends in population vitamin D status.

## 5 Conclusion

In this large, mixed-age hospital-based cohort from a single tertiary care institution in Eastern Hunan, China, vitamin D deficiency was not uniformly distributed but was concentrated in specific demographic and seasonal clusters. Adolescents, younger adults (especially women in pregnancy-related care), and individuals during the spring season exhibited the highest risk. These results underscore that even in a subtropical climate, specific patient subgroups remain vulnerable. The findings advocate for a shift from generalized to risk-based clinical management of vitamin D within hospital settings, ensuring that resources and interventions are efficiently directed toward those with the greatest need.

## Data Availability

The original contributions presented in the study are included in the article/Supplementary material, further inquiries can be directed to the corresponding author/s.
