# Supplementary material for: Prevalence and correlates of vitamin D deficiency in a mixed-age hospital-based cohort
Source: Front Public Health. 2026 Feb 19;14:1757957. doi: 10.3389/fpubh.2026.1757957 (PMC12961691; doi:10.3389/fpubh.2026.1757957)
Supplement: Supplementary file 1 [file Table_1.docx]

**Supplementary Table S1.** Seasonal and age-stratified vitamin D deficiency rates (Liuyang Hospital 2024–2025).

| **Age Group** | **Spring** | **Summer** | **Fall** | **Winter** |
| --- | --- | --- | --- | --- |
| Infants/Neonates (<1y) | 6.2 | 3.8 | 2.9 | 5.9 |
| Young Children (1-5y) | 5.1 | 2.4 | 1.8 | 3.9 |
| School-Age Children (6-12y) | 25.3 | 8.7 | 6.5 | 18.2 |
| Adolescents (13-17y) | 16.4 | 7.2 | 6.2 | 14.6 |
| Pregnancy-Focused (18-39y) | 24.8 | 10.1 | 8.7 | 20.4 |
| Older Adults (>40y) | 14.6 | 6.3 | 5.4 | 13.2 |

**Supplementary Table S2.** Correlation matrix among biochemical markers (NHANES 2009–2012). All participants with available values.

Values are Spearman’s ρ (p-value), with sample size n indicating the pairwise complete cases used for each estimate.

| **Variable 1** | **Variable 2** | **Spearman ρ** | **p-value** | **n** |
| --- | --- | --- | --- | --- |
| 25(OH)D (ng/mL) | Calcium (mg/dL) | 0.11 | <0.001 | 12115 |
| 25(OH)D (ng/mL) | Phosphorus (mg/dL) | 0.04 | <0.001 | 12113 |
| 25(OH)D (ng/mL) | Alkaline phosphatase (U/L) | -0.097 | <0.001 | 12115 |
| 25(OH)D (ng/mL) | CRP (mg/L) | -0.117 | <0.001 | 13867 |
| 25(OH)D (ng/mL) | Lead (µg/dL) | 0.016 | 0.141 | 8491 |
| 25(OH)D (ng/mL) | Cadmium (µg/L) | -0.029 | 0.01 | 8451 |
| 25(OH)D (ng/mL) | PTH (pg/mL) | -0.116 | <0.001 | 4753 |
| Calcium (mg/dL) | Phosphorus (mg/dL) | -0.064 | <0.001 | 12110 |
| Calcium (mg/dL) | Alkaline phosphatase (U/L) | -0.239 | <0.001 | 12112 |
| Calcium (mg/dL) | CRP (mg/L) | -0.127 | <0.001 | 13862 |
| Calcium (mg/dL) | Lead (µg/dL) | 0.006 | 0.623 | 8488 |
| Calcium (mg/dL) | Cadmium (µg/L) | -0.015 | 0.172 | 8448 |
| Calcium (mg/dL) | PTH (pg/mL) | -0.031 | 0.038 | 4752 |
| Phosphorus (mg/dL) | Alkaline phosphatase (U/L) | 0.167 | <0.001 | 12109 |
| Phosphorus (mg/dL) | CRP (mg/L) | 0.01 | 0.259 | 13857 |
| Phosphorus (mg/dL) | Lead (µg/dL) | 0.006 | 0.61 | 8486 |
| Phosphorus (mg/dL) | Cadmium (µg/L) | 0.017 | 0.125 | 8446 |
| Phosphorus (mg/dL) | PTH (pg/mL) | 0.031 | 0.037 | 4752 |
| Alkaline phosphatase (U/L) | CRP (mg/L) | 0.097 | <0.001 | 13862 |
| Alkaline phosphatase (U/L) | Lead (µg/dL) | 0.026 | 0.022 | 8488 |
| Alkaline phosphatase (U/L) | Cadmium (µg/L) | 0.035 | 0.001 | 8448 |
| Alkaline phosphatase (U/L) | PTH (pg/mL) | 0.088 | <0.001 | 4752 |
| CRP (mg/L) | Lead (µg/dL) | 0.038 | <0.001 | 9786 |
| CRP (mg/L) | Cadmium (µg/L) | 0.102 | <0.001 | 9746 |
| CRP (mg/L) | PTH (pg/mL) | 0.037 | 0.009 | 5744 |
| Lead (µg/dL) | Cadmium (µg/L) | 0.24 | <0.001 | 10221 |
| Lead (µg/dL) | PTH (pg/mL) | 0.019 | 0.283 | 6090 |
| Cadmium (µg/L) | PTH (pg/mL) | 0.054 | <0.001 | 6048 |
